# Crystal structure of a two-dimensional grid-type iron(II) coordination polymer: poly[[di­aqua­tetra-μ-cyanido-diargentate(I)iron(II)] *trans*-1,2-bis(pyridin-2-yl)ethyl­ene disolvate]

**DOI:** 10.1107/S1600536814016250

**Published:** 2014-07-23

**Authors:** Jintana Othong, Nanthawat Wannarit, Chaveng Pakawatchai, Sujittra Youngme

**Affiliations:** a Materials Chemistry Research Center, Department of Chemistry and Center of Excellence for Innovation in Chemistry, Faculty of Science, Khon Kaen University, Khon Kean 40002, Thailand; b Department of Chemistry, Faculty of Science and Technology, Thammasat University, Rangsit Campus, Klong Luang, Pathumthani 12121, Thailand; c Department of Chemistry, Faculty of Science, Prince of Songkla University, Hat Yai, Songkhla 90112, Thailand

**Keywords:** metal–organic framework, di­cyano­argentate(I), 1,2-bis­(pyridin-2-yl)ethyl­ene, crystal structure

## Abstract

In the title compound, {[Ag_2_Fe(CN)_4_(H_2_O)_2_]·2C_12_H_10_N_2_}_*n*_, the asymmetric unit contains one Fe^II^ cation, two water mol­ecules, two di­cyanido­argentate(I) anions and two uncoordinating 1,2-bis­(pyridin-2-yl)ethyl­ene (2,2′-bpe) mol­ecules. Each Fe^II^ atom is six-coordinated in a nearly regular octa­hedral geometry by four N atoms from di­cyanido­argentate(I) bridges and two coordinating water mol­ecules. The Fe^II^ atoms are bridged by di­cyanido­argentate(I) units to give a two-dimensional layer with square-grid spaces. The inter­grid spaces with inter­layer distance of 6.550 (2) Å are occupied by 2,2′-bpe guest mol­ecules which form O—H⋯N hydrogen bonds to the host layers. This leads to an extended three-dimensional supra­molecular architecture. The structure of the title compound is compared with some related compounds containing di­cyanido­argentate(I) ligands and N-donor organic co-ligands.

## Abstract 2

{[Fe(H_2_O)_2_{Ag(CN)_2_}_2_](2,2′-bpe)_2_}_*n*_ forms a two-dimensional grid-type structure with the organic guest mol­ecules occupying the space between adjacent grid layers. The grid layers are held together by hydrogen bonds between the organic guest mol­ecules and the host framework and gives rise to a three-dimensional supra­molecular architecture.

## Chemical context   

Metal–organic frameworks (MOFs) have attracted much attention because of their versatile topologies and dimensions. These structural properties lead to potential inter­esting applications in the filed of magnetism, sensing, porous mater­ials and catalysis (Biswas *et al.*, 2014 ▶; Horike *et al.*, 2008 ▶; Sanda *et al.*, 2013 ▶). Structural diversity in MOFs can occur as a result of various preparation methods. However, supra­molecular chemistry and topologies of MOFs are rather controlled by the nature of the metal ions and the structure of the organic ligands (Yang *et al.*, 2008 ▶).
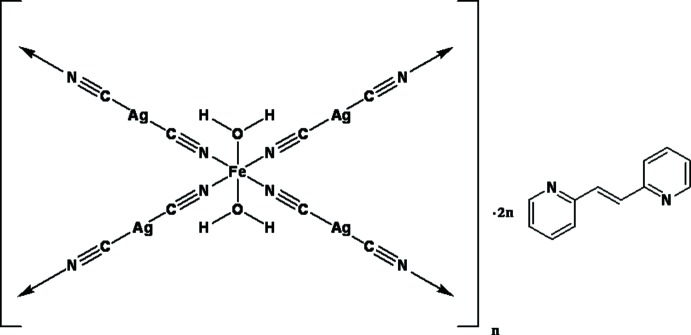


One-, two- and three-dimensional frameworks containing di­cyanido­argentate(I) and N-donor linkers such as pyrazine, 4,4′-bpy and 4,4′-bpe [bpy is bipyridineand bpe is 1,2-bis(4-pyridyl)ethylene] ligands have been studied (Soma & Iwamoto, 1996 ▶; Munoz *et al.*, 2007 ▶; Dong *et al.*, 2003 ▶). Whereas 4,4′-bpe appears to be somewhat ubiquitous in cyanido ­compounds, its cousin 2,2′-bpe is not very often used, which led us to prepare a di­cyanido­argentate(I) compound with a 2,2′-bpe ligand. In this communication, we report the synthesis and crystal structure of a three-dimensional supra­molecular framework of {[Ag_2_Fe(CN)_4_(H_2_O)_2_]·2C_12_H_10_N_2_}_*n*_, (I).

## Structural commentary   

**Figure 1:**
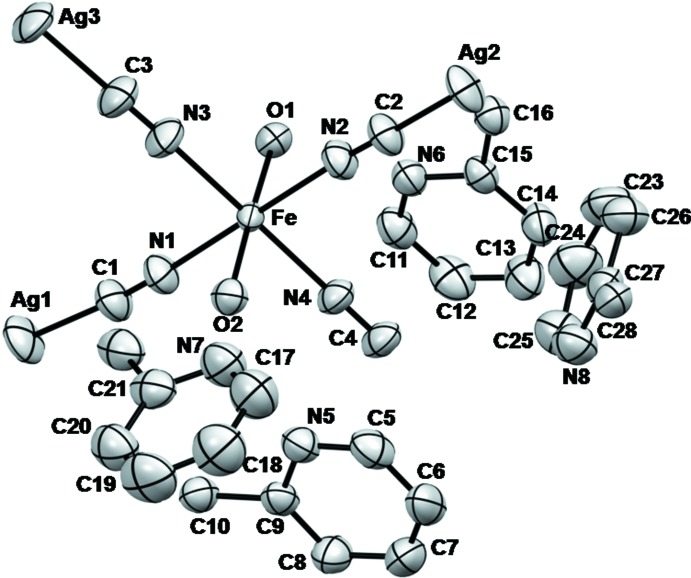
A view of the asymmetric unit in (I), showing displacement ellipsoids at the 50% probability level and the atom-numbering scheme. H atoms have been omitted for clarity.

**Figure 2:**
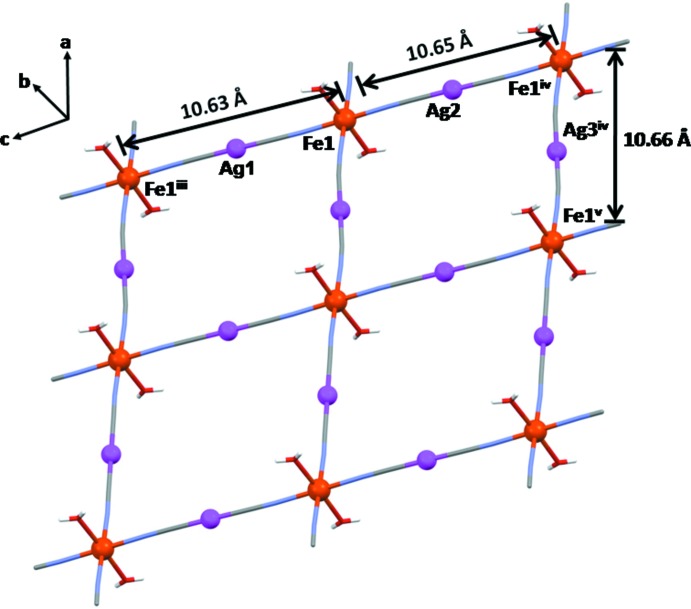
A view of the square grid of (I) in the *ac* plane; the 2,2′-bpe mol­ecules have been omitted. [Symmetry codes: (iii) −*x* + 1, −*y* + 2, *z*; (iv) −*x*, −*y* + 1, −*z* + 1; (v) −*x* + 1, −*y*, −*z* + 1.]

The asymmetric unit consists of one Fe^II^ atom, two di­cyan­ido­argentate(I) ligands, two water mol­ecules and two uncoord­inating 2,2′-bpe mol­ecules (Fig. 1 ▶). Ag1 and Ag2 are situated on inversion centres. The dicyanidoargentate(I) ligands link Fe^II^ atoms into an infinite two-dimensional layer network with a nearly square-grid geometry of 10.66 × 10.64 Å^2^ (Fig. 2 ▶). The Fe^II^ ion is six-cooordinated in a nearly regular octa­hedral geometry by four N atoms from four di­cyanido­argentate(I) ligands and two water mol­ecules.

## Supra­molecular features   

**Figure 3:**
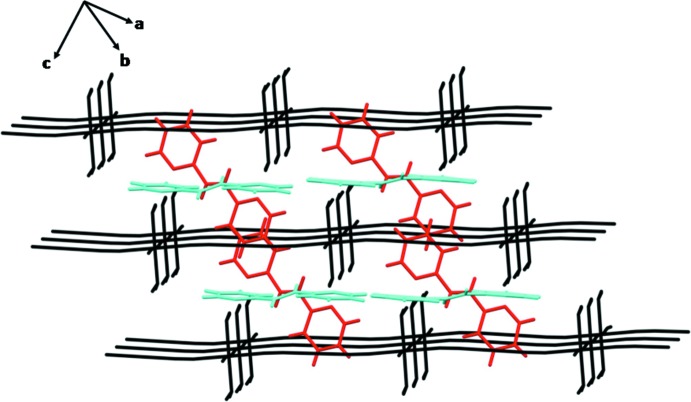
2,2′-Bpe in parallel (blue) and non-parallel (red) fashion between adjacent layers.

**Figure 4:**
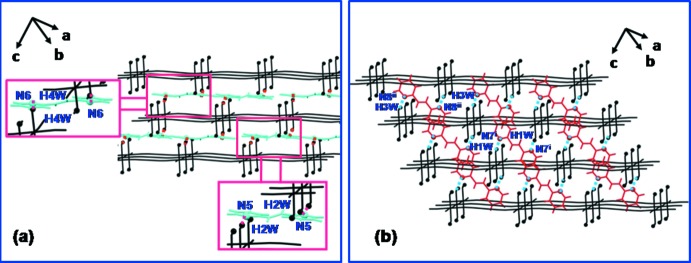
A fragment of the three-dimensional supra­molecular framework *via* N⋯H—O hydrogen-bonding inter­actions between (*a*) parallel 2,2′-bpe and coordinating water mol­ecules (dashed lines), and (*b*) non-parallel 2,2′-bpe and coordinating water mol­ecules (dashed lines). [Symmetry codes: (i) *x* − 1, *y*, *z*; (ii) *x*, *y* + 1, *z*.]

Four independent 2,2′-bpe mol­ecules are located between adjacent grid layers of which two are parallel (blue) to the grid layers and two non-parallel (red) (Fig. 3 ▶). The inter­layer distance is 6.550 (2) Å. The two parallel 2,2′-bpe ligands form hydrogen bonds to the host layer (O1—H2*W*⋯N5 = 2.07 Å and O2–H4*W*⋯N6 = 2.09 Å) (Fig. 4 ▶ *a*), while the other two arrange themselves across the host layer to form also hydrogen bonds (O1—H1*W*⋯N7 = 2.14 Å and O2—H3*W*⋯N8 = 2.15 Å) (Fig. 4 ▶ *b*) to the host layers. These hydrogen bonds generate an extended three-dimensional supra­molecular framework.

## Database survey   

The two-dimensional structure of (I) was found to be different from other closely related compounds. In the structure of [Cd(imH)_4_[Ag(CN)_2_]_2_]_*n*_ (imH = imidazole), a one-dimensional chain *via* bridging di­cyanido­argentate(I) is found, while all imidazole mol­ecules act as a terminal ligand (Takayoshi & Toschitake, 1996 ▶). In addition, the two-dimensional framework of [Fe(3-Fpy)_2_[Ag(CN)_2_]_2_]_*n*_ (3-Fpy = 3-fluoro­pyridine) consists of four cyanide moieties occupying the equatorial positions generating a square grid-type structure similar to that of the title compound, while the axial positions are occupied by two terminal 3-Fpy ligands instead of two water mol­ecules in (I) (Munoz *et al.*, 2007 ▶). When the terminal ligands such as imH and 3-Fpy are replaced by N-donor linkers such as pyrazine, 4,4′-bpy and 4,4′-bpe, three-dimensional inter­penetrating frameworks are obtained, as in {[Fe(pz)[Ag(CN)_2_]_2_].pz}_*n*_ (pz = pyrazine), [Mn(4,4′-bpy)_2_[Ag(CN)_2_]_2_]_*n*_, [Fe(4,4′-bpy)_2_[Ag(CN)_2_]_2_]_*n*_ and [Fe(bpe)_2_[Ag(CN)_2_]_2_]_*n*_ (Niel *et al.*, 2002 ▶; Dong *et al.*, 2003 ▶). The last compound contains bpe bridges, while in the title compound 2,2′-bpe behaves as the organic guest mol­ecules in the lattice. This could be the result of the difference in the N-donor position.

## Synthesis and crystallization   

An aqueous solution (5 ml) of K[Ag(CN)_2_] (0.0995 g, 0.5 mmol) was added dropwise to an MeOH–H_2_O mixed solution (1:1 *v*/*v*, 10 ml) of (NH_4_)_2_[Fe(SO_4_)_2_]·6H_2_O (0.0980 g, 0.25 mmol) and 2,2′-bpe (0.0911 g, 0.5 mmol) at room temperature. After filtration and slow evaporation for 1 d, yellow crystals were obtained.

## Refinement details   

Crystal data, data collection and structure refinement details are summarized in Table 1 ▶. C-bound H atoms were positioned geometrically and included as riding atoms, with aromatic C—H = 0.93 Å and *U_iso_*(H) = 1.2*U_eq_*(C). Water H atoms were located in difference Fourier maps and refined isotropically.

## Supplementary Material

Crystal structure: contains datablock(s) I. DOI: 10.1107/S1600536814016250/vn2085sup1.cif

Structure factors: contains datablock(s) I. DOI: 10.1107/S1600536814016250/vn2085Isup2.hkl

CCDC reference: 1013775

Additional supporting information:  crystallographic information; 3D view; checkCIF report

## Figures and Tables

**Table 1:** Selected bond lengths (Å)

Fe—O1	2.1365 (15)	Fe—N4	2.1489 (16)
Fe—O2	2.1392 (16)	Fe—N2	2.1522 (16)
Fe—N1	2.1440 (17)	Fe—N3	2.1539 (17)

**Table 2:** Hydrogen-bond geometry (Å, °)

*D*—H⋯*A*	*D*—H	H⋯*A*	*D*⋯*A*	*D*—H⋯*A*
O1—H2*W*⋯N5	0.76 (3)	2.07 (3)	2.829 (2)	174 (2)
O2—H4*W*⋯N6	0.73 (3)	2.09 (3)	2.823 (3)	174 (3)
O1—H1*W*⋯N7^i^	0.75 (3)	2.14 (3)	2.870 (3)	164
O2—H3*W*⋯N8^ii^	0.74 (3)	2.15 (3)	2.868 (3)	162

Symmetry codes: (i) 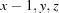; (ii) 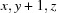.

**Table 3:** Experimental details

Crystal data	
Chemical formula	[Ag_2_Fe(CN)_4_(H_2_O)_2_]·2C_12_H_10_N_2_
*M*_r_	776.14
Crystal system, space group	Triclinic, *P* 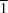
Temperature (K)	293
*a*, *b*, *c* (Å)	9.2078 (4), 9.8558 (5), 18.9029 (9)
α, β, γ (°)	77.667 (1), 77.507 (1), 67.900 (1)
*V* (Å^3^)	1535.11 (13)
*Z*	2
Radiation type	Mo *K*α
μ (mm^−1^)	1.77
Crystal size (mm)	0.43 × 0.11 × 0.09

Data collection	
Diffractometer	Bruker SMART CCD area detector
Absorption correction	Multi-scan (*SADABS*; Bruker, 2007 ▶)
*T*_min_, *T*_max_	0.684, 1.000
No. of measured, independent and observed [*I* > 2σ(*I*)] reflections	21143, 7389, 5865
*R*_int_	0.024
(sin θ/λ)_max_ (Å^−1^)	0.661

Refinement	
*R*[*F* ^2^ > 2σ(*F* ^2^)], *wR*(*F* ^2^), *S*	0.029, 0.073, 1.03
No. of reflections	7389
No. of parameters	389
H-atom treatment	H atoms treated by a mixture of independent and constrained refinement
Δρ_max_, Δρ_min_ (e Å^−3^)	0.32, −0.37

Computer programs: *SMART* and *SAINT* (Bruker, 2007 ▶), *SHELXS97* and *SHELXL97* (Sheldrick, 2008 ▶), *Mercury* (Macrae *et al.*, 2008 ▶) and *publCIF* (Westrip, 2010 ▶).

## supplementary crystallographic information

### Crystal data

**Table:**

[Ag_2_Fe(CN)_4_(H_2_O)_2_]·2C_12_H_10_N_2_	*V* = 1535.11 (13) Å^3^
*M**_r_* = 776.14	*Z* = 2
Triclinic, *P*1	*F*(000) = 768
Hall symbol: -P 1	776.14
*a* = 9.2078 (4) Å	*D*_x_ = 1.679 Mg m^−^^3^
*b* = 9.8558 (5) Å	Mo *K*α radiation, λ = 0.71073 Å
*c* = 18.9029 (9) Å	µ = 1.77 mm^−^^1^
α = 77.667 (1)°	*T* = 293 K
β = 77.507 (1)°	Block, yellow
γ = 67.900 (1)°	0.43 × 0.11 × 0.09 mm

### Data collection

**Table:**

Bruker SMART CCD area-detector diffractometer	7389 independent reflections
Radiation source: fine-focus sealed tube	5865 reflections with *I* > 2σ(*I*)
Graphite monochromator	*R*_int_ = 0.024
phi and ω scans	θ_max_ = 28.0°, θ_min_ = 1.1°
Absorption correction: multi-scan (*SADABS*; Bruker, 2007)	*h* = −12→12
*T*_min_ = 0.684, *T*_max_ = 1.000	*k* = −13→13
21143 measured reflections	*l* = −24→24

### Refinement

**Table:**

Refinement on *F*^2^	Primary atom site location: structure-invariant direct methods
Least-squares matrix: full	Secondary atom site location: difference Fourier map
*R*[*F*^2^ > 2σ(*F*^2^)] = 0.029	Hydrogen site location: inferred from neighbouring sites
*wR*(*F*^2^) = 0.073	H atoms treated by a mixture of independent and constrained refinement
*S* = 1.03	*w* = 1/[σ^2^(*F*_o_^2^) + (0.0324*P*)^2^ + 0.1941*P*] where *P* = (*F*_o_^2^ + 2*F*_c_^2^)/3
7389 reflections	(Δ/σ)_max_ = 0.001
389 parameters	Δρ_max_ = 0.32 e Å^−^^3^
0 restraints	Δρ_min_ = −0.37 e Å^−^^3^

### Special details

**Table:**

Geometry. All e.s.d.'s (except the e.s.d. in the dihedral angle between two l.s. planes) are estimated using the full covariance matrix. The cell e.s.d.'s are taken into account individually in the estimation of e.s.d.'s in distances, angles and torsion angles; correlations between e.s.d.'s in cell parameters are only used when they are defined by crystal symmetry. An approximate (isotropic) treatment of cell e.s.d.'s is used for estimating e.s.d.'s involving l.s. planes.
Refinement. Refinement of *F*^2^ against ALL reflections. The weighted *R*-factor *wR* and goodness of fit *S* are based on *F*^2^, conventional *R*-factors *R* are based on *F*, with *F* set to zero for negative *F*^2^. The threshold expression of *F*^2^ > σ(*F*^2^) is used only for calculating *R*-factors(gt) *etc*. and is not relevant to the choice of reflections for refinement. *R*-factors based on *F*^2^ are statistically about twice as large as those based on *F*, and *R*- factors based on ALL data will be even larger.

### Fractional atomic coordinates and isotropic or equivalent isotropic displacement parameters (Å^2^)

**Table:**

	*x*	*y*	*z*	*U*_iso_*/*U*_eq_	
Ag3	−0.235709 (19)	1.239825 (17)	0.249406 (10)	0.06308 (7)	
Ag2	0.0000	0.5000	0.5000	0.05956 (8)	
Ag1	0.5000	1.0000	0.0000	0.06538 (9)	
Fe	0.26653 (3)	0.74083 (2)	0.251508 (12)	0.02989 (7)	
O1	0.15978 (19)	0.64325 (19)	0.19630 (8)	0.0437 (3)	
O2	0.3821 (2)	0.83190 (18)	0.30581 (9)	0.0440 (3)	
N3	0.0604 (2)	0.9394 (2)	0.25270 (11)	0.0562 (5)	
N2	0.1689 (2)	0.64872 (19)	0.35630 (9)	0.0476 (4)	
N1	0.3633 (2)	0.83204 (19)	0.14683 (9)	0.0497 (4)	
N4	0.4711 (2)	0.54179 (18)	0.24955 (10)	0.0453 (4)	
N5	0.3585 (2)	0.37522 (19)	0.14391 (9)	0.0472 (4)	
N6	0.6565 (2)	0.62355 (18)	0.36001 (9)	0.0446 (4)	
N7	0.9350 (3)	0.7412 (3)	0.09581 (12)	0.0717 (6)	
C26	0.2054 (3)	0.1175 (3)	0.52221 (14)	0.0675 (7)	
H26	0.1402	0.1178	0.5673	0.081*	
C3	−0.0459 (3)	1.0458 (3)	0.25155 (14)	0.0618 (6)	
C2	0.1135 (3)	0.5943 (2)	0.40815 (11)	0.0508 (5)	
C1	0.4140 (3)	0.8860 (3)	0.09364 (11)	0.0541 (5)	
C4	0.5760 (2)	0.4344 (2)	0.24875 (12)	0.0494 (5)	
C5	0.3629 (3)	0.2536 (3)	0.19190 (12)	0.0577 (6)	
H5	0.2794	0.2601	0.2304	0.069*	
C6	0.4827 (3)	0.1197 (3)	0.18812 (13)	0.0653 (7)	
H6	0.4789	0.0369	0.2220	0.078*	
C7	0.6076 (3)	0.1114 (3)	0.13335 (13)	0.0676 (7)	
H7	0.6930	0.0232	0.1303	0.081*	
C8	0.6063 (3)	0.2340 (2)	0.08276 (12)	0.0569 (6)	
H8	0.6908	0.2295	0.0450	0.068*	
C9	0.4787 (2)	0.3644 (2)	0.08807 (10)	0.0422 (4)	
C10	0.4628 (2)	0.4983 (2)	0.03421 (11)	0.0458 (5)	
H10	0.3953	0.5880	0.0492	0.055*	
C11	0.7884 (3)	0.6068 (2)	0.31163 (11)	0.0534 (5)	
H11	0.7929	0.6865	0.2756	0.064*	
C12	0.9174 (3)	0.4796 (3)	0.31178 (13)	0.0596 (6)	
H12	1.0074	0.4736	0.2771	0.072*	
C13	0.9117 (3)	0.3612 (3)	0.36401 (13)	0.0610 (6)	
H13	0.9963	0.2719	0.3646	0.073*	
C14	0.7776 (3)	0.3769 (2)	0.41567 (12)	0.0516 (5)	
H14	0.7718	0.2984	0.4521	0.062*	
C15	0.6523 (2)	0.5092 (2)	0.41323 (10)	0.0405 (4)	
C16	0.5075 (2)	0.5375 (2)	0.46672 (11)	0.0442 (5)	
H16	0.4180	0.6149	0.4528	0.053*	
C17	0.8716 (4)	0.6370 (3)	0.10434 (16)	0.0836 (8)	
H17	0.8639	0.5809	0.1504	0.100*	
C18	0.8173 (4)	0.6069 (4)	0.05021 (19)	0.0864 (9)	
H18	0.7721	0.5338	0.0590	0.104*	
C19	0.8315 (4)	0.6881 (4)	−0.01761 (19)	0.0913 (10)	
H19	0.7987	0.6689	−0.0564	0.110*	
C20	0.8947 (3)	0.7982 (3)	−0.02814 (15)	0.0754 (7)	
H20	0.9050	0.8541	−0.0741	0.091*	
C21	0.9428 (3)	0.8251 (3)	0.03048 (14)	0.0603 (6)	
C22	1.0045 (3)	0.9443 (3)	0.02706 (13)	0.0649 (7)	
H22	1.0535	0.9411	0.0658	0.078*	
C23	0.3203 (4)	0.1815 (3)	0.50884 (18)	0.0817 (9)	
H23	0.3330	0.2257	0.5448	0.098*	
C24	0.4143 (4)	0.1796 (3)	0.44306 (17)	0.0782 (8)	
H24	0.4900	0.2252	0.4321	0.094*	
C25	0.3943 (3)	0.1080 (3)	0.39311 (15)	0.0762 (7)	
H25	0.4617	0.1032	0.3486	0.091*	
N8	0.2859 (2)	0.0455 (2)	0.40410 (11)	0.0627 (5)	
C27	0.1880 (3)	0.0527 (2)	0.46773 (12)	0.0534 (5)	
C28	0.0623 (3)	−0.0088 (2)	0.47413 (12)	0.0574 (6)	
H28	0.0714	−0.0651	0.4386	0.069*	
H1W	0.096 (3)	0.684 (2)	0.1727 (12)	0.044 (7)*	
H2W	0.211 (3)	0.574 (3)	0.1795 (13)	0.062 (8)*	
H3W	0.339 (3)	0.887 (3)	0.3313 (13)	0.053 (8)*	
H4W	0.450 (3)	0.779 (3)	0.3230 (14)	0.062 (9)*	

### Atomic displacement parameters (Å^2^)

**Table:**

	*U*^11^	*U*^22^	*U*^33^	*U*^12^	*U*^13^	*U*^23^
Ag3	0.04673 (11)	0.03995 (10)	0.08458 (15)	0.01369 (7)	−0.01787 (10)	−0.01697 (9)
Ag2	0.06824 (17)	0.07681 (18)	0.03192 (12)	−0.03820 (14)	0.00292 (11)	0.00933 (11)
Ag1	0.08051 (19)	0.07607 (18)	0.03474 (13)	−0.03990 (15)	0.00655 (12)	0.01030 (12)
Fe	0.02927 (13)	0.02553 (12)	0.02578 (13)	−0.00351 (10)	0.00002 (10)	−0.00020 (9)
O1	0.0401 (8)	0.0444 (8)	0.0442 (8)	−0.0085 (7)	−0.0104 (7)	−0.0089 (7)
O2	0.0484 (9)	0.0357 (8)	0.0442 (9)	−0.0082 (7)	−0.0085 (7)	−0.0083 (7)
N3	0.0452 (10)	0.0412 (10)	0.0621 (12)	0.0063 (8)	−0.0073 (9)	−0.0065 (9)
N2	0.0514 (10)	0.0524 (10)	0.0336 (9)	−0.0206 (8)	0.0007 (8)	0.0023 (7)
N1	0.0570 (11)	0.0512 (10)	0.0345 (9)	−0.0208 (8)	0.0017 (8)	0.0025 (8)
N4	0.0391 (9)	0.0361 (8)	0.0541 (10)	0.0004 (7)	−0.0125 (8)	−0.0127 (7)
N5	0.0513 (10)	0.0511 (10)	0.0395 (9)	−0.0182 (8)	−0.0043 (8)	−0.0090 (8)
N6	0.0517 (10)	0.0450 (9)	0.0375 (9)	−0.0177 (8)	−0.0080 (8)	−0.0038 (7)
N7	0.0780 (15)	0.0757 (15)	0.0653 (14)	−0.0183 (12)	−0.0273 (12)	−0.0168 (12)
C26	0.0747 (17)	0.0638 (15)	0.0627 (16)	−0.0141 (13)	−0.0104 (13)	−0.0247 (12)
C3	0.0515 (13)	0.0444 (12)	0.0731 (16)	0.0079 (10)	−0.0148 (12)	−0.0148 (11)
C2	0.0566 (13)	0.0598 (13)	0.0332 (11)	−0.0254 (11)	0.0003 (9)	0.0016 (9)
C1	0.0663 (14)	0.0579 (13)	0.0349 (11)	−0.0274 (11)	0.0017 (10)	0.0015 (10)
C4	0.0424 (11)	0.0383 (10)	0.0636 (14)	0.0011 (9)	−0.0189 (10)	−0.0162 (10)
C5	0.0677 (15)	0.0672 (15)	0.0414 (12)	−0.0327 (13)	0.0031 (11)	−0.0089 (11)
C6	0.103 (2)	0.0478 (13)	0.0464 (13)	−0.0329 (14)	−0.0056 (13)	−0.0022 (10)
C7	0.090 (2)	0.0433 (13)	0.0573 (15)	−0.0114 (13)	−0.0043 (14)	−0.0107 (11)
C8	0.0627 (14)	0.0482 (12)	0.0491 (13)	−0.0138 (11)	0.0050 (11)	−0.0087 (10)
C9	0.0516 (12)	0.0421 (10)	0.0383 (11)	−0.0199 (9)	−0.0060 (9)	−0.0105 (8)
C10	0.0500 (12)	0.0423 (11)	0.0462 (11)	−0.0165 (9)	−0.0036 (9)	−0.0108 (9)
C11	0.0642 (14)	0.0580 (13)	0.0425 (12)	−0.0310 (12)	−0.0055 (10)	−0.0008 (10)
C12	0.0448 (12)	0.0818 (17)	0.0503 (13)	−0.0248 (12)	−0.0002 (10)	−0.0073 (12)
C13	0.0452 (12)	0.0687 (16)	0.0562 (14)	−0.0060 (11)	−0.0112 (11)	−0.0042 (12)
C14	0.0489 (12)	0.0544 (12)	0.0428 (12)	−0.0122 (10)	−0.0103 (10)	0.0036 (10)
C15	0.0431 (10)	0.0472 (11)	0.0352 (10)	−0.0190 (9)	−0.0102 (8)	−0.0040 (8)
C16	0.0423 (11)	0.0463 (11)	0.0430 (11)	−0.0141 (9)	−0.0099 (9)	−0.0034 (9)
C17	0.095 (2)	0.084 (2)	0.0752 (19)	−0.0251 (17)	−0.0280 (17)	−0.0129 (16)
C18	0.084 (2)	0.092 (2)	0.096 (2)	−0.0307 (17)	−0.0325 (18)	−0.0203 (19)
C19	0.086 (2)	0.109 (3)	0.092 (2)	−0.0224 (19)	−0.0425 (19)	−0.034 (2)
C20	0.0716 (17)	0.089 (2)	0.0643 (17)	−0.0151 (15)	−0.0242 (14)	−0.0176 (15)
C21	0.0434 (12)	0.0682 (15)	0.0641 (15)	−0.0006 (11)	−0.0167 (11)	−0.0247 (13)
C22	0.0491 (13)	0.0804 (18)	0.0590 (16)	−0.0037 (13)	−0.0168 (12)	−0.0234 (12)
C23	0.094 (2)	0.0747 (19)	0.088 (2)	−0.0216 (17)	−0.0221 (18)	−0.0403 (17)
C24	0.083 (2)	0.0746 (18)	0.090 (2)	−0.0330 (16)	−0.0194 (17)	−0.0229 (16)
C25	0.0807 (19)	0.089 (2)	0.0634 (17)	−0.0311 (16)	−0.0094 (14)	−0.0180 (14)
N8	0.0666 (13)	0.0675 (13)	0.0563 (12)	−0.0171 (11)	−0.0154 (10)	−0.0189 (10)
C27	0.0594 (13)	0.0383 (11)	0.0583 (14)	−0.0029 (10)	−0.0216 (11)	−0.0111 (10)
C28	0.0711 (16)	0.0411 (11)	0.0533 (14)	−0.0038 (11)	−0.0189 (11)	−0.0124 (10)

### Geometric parameters (Å, º)

**Table:**

Ag3—C4^i^	2.0449 (19)	C8—H8	0.9300
Ag3—C3	2.048 (2)	C9—C10	1.465 (3)
Ag2—C2^ii^	2.056 (2)	C10—C10^v^	1.326 (4)
Ag2—C2	2.056 (2)	C10—H10	0.9300
Ag1—C1	2.058 (2)	C11—C12	1.364 (3)
Ag1—C1^iii^	2.058 (2)	C11—H11	0.9300
Fe—O1	2.1365 (15)	C12—C13	1.368 (3)
Fe—O2	2.1392 (16)	C12—H12	0.9300
Fe—N1	2.1440 (17)	C13—C14	1.378 (3)
Fe—N4	2.1489 (16)	C13—H13	0.9300
Fe—N2	2.1522 (16)	C14—C15	1.377 (3)
Fe—N3	2.1539 (17)	C14—H14	0.9300
O1—H1W	0.75 (2)	C15—C16	1.462 (3)
O1—H2W	0.76 (2)	C16—C16^vi^	1.324 (4)
O2—H3W	0.74 (2)	C16—H16	0.9300
O2—H4W	0.73 (3)	C17—C18	1.360 (4)
N3—C3	1.133 (3)	C17—H17	0.9300
N2—C2	1.129 (3)	C18—C19	1.367 (4)
N1—C1	1.126 (3)	C18—H18	0.9300
N4—C4	1.133 (2)	C19—C20	1.374 (4)
N5—C5	1.333 (3)	C19—H19	0.9300
N5—C9	1.343 (2)	C20—C21	1.386 (3)
N6—C11	1.332 (3)	C20—H20	0.9300
N6—C15	1.347 (2)	C21—C22	1.471 (4)
N7—C17	1.327 (3)	C22—C22^vii^	1.322 (5)
N7—C21	1.336 (3)	C22—H22	0.9300
C26—C23	1.378 (4)	C23—C24	1.352 (4)
C26—C27	1.387 (3)	C23—H23	0.9300
C26—H26	0.9300	C24—C25	1.371 (3)
C4—Ag3^iv^	2.0449 (19)	C24—H24	0.9300
C5—C6	1.369 (3)	C25—N8	1.318 (3)
C5—H5	0.9300	C25—H25	0.9300
C6—C7	1.360 (3)	N8—C27	1.335 (3)
C6—H6	0.9300	C27—C28	1.469 (3)
C7—C8	1.369 (3)	C28—C28^viii^	1.320 (5)
C7—H7	0.9300	C28—H28	0.9300
C8—C9	1.382 (3)		
			
C4^i^—Ag3—C3	179.00 (8)	C10^v^—C10—H10	117.5
C2^ii^—Ag2—C2	180.000 (1)	C9—C10—H10	117.5
C1—Ag1—C1^iii^	180.00 (16)	N6—C11—C12	123.8 (2)
O1—Fe—O2	177.77 (6)	N6—C11—H11	118.1
O1—Fe—N1	88.80 (6)	C12—C11—H11	118.1
O2—Fe—N1	90.70 (7)	C11—C12—C13	118.7 (2)
O1—Fe—N4	88.18 (7)	C11—C12—H12	120.7
O2—Fe—N4	89.65 (7)	C13—C12—H12	120.7
N1—Fe—N4	90.30 (7)	C12—C13—C14	118.5 (2)
O1—Fe—N2	90.90 (6)	C12—C13—H13	120.7
O2—Fe—N2	89.60 (6)	C14—C13—H13	120.7
N1—Fe—N2	179.69 (6)	C15—C14—C13	119.9 (2)
N4—Fe—N2	89.68 (7)	C15—C14—H14	120.0
O1—Fe—N3	91.17 (7)	C13—C14—H14	120.0
O2—Fe—N3	91.00 (7)	N6—C15—C14	121.16 (19)
N1—Fe—N3	89.61 (7)	N6—C15—C16	115.02 (17)
N4—Fe—N3	179.35 (6)	C14—C15—C16	123.81 (18)
N2—Fe—N3	90.41 (7)	C16^vi^—C16—C15	125.7 (2)
Fe—O1—H1W	126.2 (17)	C16^vi^—C16—H16	117.2
Fe—O1—H2W	119.1 (19)	C15—C16—H16	117.2
H1W—O1—H2W	106 (2)	N7—C17—C18	124.3 (3)
Fe—O2—H3W	123.3 (19)	N7—C17—H17	117.9
Fe—O2—H4W	116 (2)	C18—C17—H17	117.9
H3W—O2—H4W	106 (3)	C17—C18—C19	117.6 (3)
C3—N3—Fe	178.0 (2)	C17—C18—H18	121.2
C2—N2—Fe	174.23 (18)	C19—C18—H18	121.2
C1—N1—Fe	176.22 (19)	C18—C19—C20	119.7 (3)
C4—N4—Fe	177.91 (19)	C18—C19—H19	120.2
C5—N5—C9	117.53 (19)	C20—C19—H19	120.2
C11—N6—C15	117.80 (18)	C19—C20—C21	119.2 (3)
C17—N7—C21	118.4 (2)	C19—C20—H20	120.4
C23—C26—C27	119.3 (3)	C21—C20—H20	120.4
C23—C26—H26	120.4	N7—C21—C20	120.8 (3)
C27—C26—H26	120.4	N7—C21—C22	114.9 (2)
N3—C3—Ag3	179.1 (2)	C20—C21—C22	124.3 (3)
N2—C2—Ag2	176.6 (2)	C22^vii^—C22—C21	124.8 (3)
N1—C1—Ag1	175.4 (2)	C22^vii^—C22—H22	117.6
N4—C4—Ag3^iv^	178.9 (2)	C21—C22—H22	117.6
N5—C5—C6	124.1 (2)	C24—C23—C26	119.5 (3)
N5—C5—H5	118.0	C24—C23—H23	120.2
C6—C5—H5	118.0	C26—C23—H23	120.2
C7—C6—C5	118.0 (2)	C23—C24—C25	117.7 (3)
C7—C6—H6	121.0	C23—C24—H24	121.1
C5—C6—H6	121.0	C25—C24—H24	121.1
C6—C7—C8	119.4 (2)	N8—C25—C24	124.3 (3)
C6—C7—H7	120.3	N8—C25—H25	117.9
C8—C7—H7	120.3	C24—C25—H25	117.9
C7—C8—C9	119.7 (2)	C25—N8—C27	118.2 (2)
C7—C8—H8	120.2	N8—C27—C26	120.8 (2)
C9—C8—H8	120.2	N8—C27—C28	115.41 (19)
N5—C9—C8	121.18 (19)	C26—C27—C28	123.8 (2)
N5—C9—C10	115.36 (18)	C28^viii^—C28—C27	125.7 (3)
C8—C9—C10	123.46 (19)	C28^viii^—C28—H28	117.2
C10^v^—C10—C9	125.1 (2)	C27—C28—H28	117.2
			
O1—Fe—N3—C3	−98 (7)	C7—C8—C9—C10	−176.4 (2)
O2—Fe—N3—C3	82 (7)	N5—C9—C10—C10^v^	−158.2 (3)
N1—Fe—N3—C3	−9 (7)	C8—C9—C10—C10^v^	21.1 (4)
N4—Fe—N3—C3	−91 (9)	C15—N6—C11—C12	1.8 (3)
N2—Fe—N3—C3	172 (7)	N6—C11—C12—C13	0.6 (4)
O1—Fe—N2—C2	6.4 (19)	C11—C12—C13—C14	−2.0 (4)
O2—Fe—N2—C2	−171.4 (19)	C12—C13—C14—C15	1.0 (4)
N1—Fe—N2—C2	5 (14)	C11—N6—C15—C14	−2.8 (3)
N4—Fe—N2—C2	−81.7 (19)	C11—N6—C15—C16	176.90 (17)
N3—Fe—N2—C2	97.6 (19)	C13—C14—C15—N6	1.5 (3)
O1—Fe—N1—C1	153 (3)	C13—C14—C15—C16	−178.2 (2)
O2—Fe—N1—C1	−29 (3)	N6—C15—C16—C16^vi^	−159.6 (3)
N4—Fe—N1—C1	−119 (3)	C14—C15—C16—C16^vi^	20.1 (4)
N2—Fe—N1—C1	155 (12)	C21—N7—C17—C18	1.7 (4)
N3—Fe—N1—C1	62 (3)	N7—C17—C18—C19	1.1 (5)
O1—Fe—N4—C4	−34 (5)	C17—C18—C19—C20	−1.9 (5)
O2—Fe—N4—C4	146 (5)	C18—C19—C20—C21	0.0 (5)
N1—Fe—N4—C4	−123 (5)	C17—N7—C21—C20	−3.8 (4)
N2—Fe—N4—C4	57 (5)	C17—N7—C21—C22	176.0 (2)
N3—Fe—N4—C4	−41 (9)	C19—C20—C21—N7	3.0 (4)
Fe—N3—C3—Ag3	−52 (20)	C19—C20—C21—C22	−176.8 (3)
C4^i^—Ag3—C3—N3	−52 (18)	N7—C21—C22—C22^vii^	−167.0 (3)
Fe—N2—C2—Ag2	−54 (5)	C20—C21—C22—C22^vii^	12.9 (5)
C2^ii^—Ag2—C2—N2	95 (100)	C27—C26—C23—C24	−0.2 (4)
Fe—N1—C1—Ag1	−20 (6)	C26—C23—C24—C25	−2.4 (5)
C1^iii^—Ag1—C1—N1	−164 (100)	C23—C24—C25—N8	2.5 (5)
Fe—N4—C4—Ag3^iv^	−78 (13)	C24—C25—N8—C27	0.4 (4)
C9—N5—C5—C6	0.7 (3)	C25—N8—C27—C26	−3.2 (3)
N5—C5—C6—C7	2.2 (4)	C25—N8—C27—C28	175.4 (2)
C5—C6—C7—C8	−2.6 (4)	C23—C26—C27—N8	3.2 (4)
C6—C7—C8—C9	0.2 (4)	C23—C26—C27—C28	−175.4 (2)
C5—N5—C9—C8	−3.3 (3)	N8—C27—C28—C28^viii^	−167.6 (3)
C5—N5—C9—C10	176.02 (18)	C26—C27—C28—C28^viii^	11.0 (4)
C7—C8—C9—N5	2.9 (3)		

Symmetry codes: (i) *x*−1, *y*+1, *z*; (ii) −*x*, −*y*+1, −*z*+1; (iii) −*x*+1, −*y*+2, −*z*; (iv) *x*+1, *y*−1, *z*; (v) −*x*+1, −*y*+1, −*z*; (vi) −*x*+1, −*y*+1, −*z*+1; (vii) −*x*+2, −*y*+2, −*z*; (viii) −*x*, −*y*, −*z*+1.

### Hydrogen-bond geometry (Å, º)

**Table:**

*D*—H···*A*	*D*—H	H···*A*	*D*···*A*	*D*—H···*A*
O1—H2*W*···N5	0.76 (3)	2.07 (3)	2.829 (2)	174 (2)
O2—H4*W*···N6	0.73 (3)	2.09 (3)	2.823 (3)	174 (3)
O1—H1*W*···N7^ix^	0.75 (3)	2.14 (3)	2.870 (3)	164
O2—H3*W*···N8^x^	0.74 (3)	2.15 (3)	2.868 (3)	162

Symmetry codes: (ix) *x*−1, *y*, *z*; (x) *x*, *y*+1, *z*.
